# Fiscal policies in the Federation of Bosnia and Herzegovina: are spending or revenue measures more effective?

**DOI:** 10.1007/s10663-022-09562-9

**Published:** 2023-01-09

**Authors:** Klaus Weyerstrass, Rijad Kovac

**Affiliations:** 1 Institute for Advanced Studies Vienna, Institute for Advanced Studies, Research Group Energy, Environment, and Sustainable Economic Structures, Josefstädter Straße 39, 1080 Vienna, Austria; 2 Federal Institute for Development Programming, 71000 Sarajevo, Bosnia and Herzegovina

**Keywords:** Macroeconomics, Stabilisation policy, Fiscal policy, Tax policy, Public expenditure, Bosnia and Herzegovina, E62, E17, E37

## Abstract

We examine the effectiveness of different fiscal policies in the Federation of Bosnia and Herzegovina (FBiH). For this purpose, we use a structural macroeconomic model for the FBiH. In this model, GDP in the Federation is influenced by world demand and by domestic demand in the Federation. Domestic demand comprises consumption of private households, public consumption, and gross fixed capital formation. Employment depends positively on GDP and negatively on the tax wedge, i.e., the net wage plus social security contribution rates (including the unemployment insurance), and the personal income tax rate in the Federation. The latter allows the analysis of the impact of changes in social security contribution rates or in the income tax rate in the Federation of Bosnia and Herzegovina. The following Federation-specific policy instruments are implemented in the model for the FBiH: Pension funds contribution rate in FBiH; contribution rate for health insurance in FBiH; contribution rate for the unemployment insurance in FBiH; benefits from social security; direct tax rates (income tax rate, corporate tax rate); public consumption in FBiH. Our results show that policy measures that reduce the tax wedge on labour income are highly effective in stimulating employment. Due to the large elasticity of imports with respect to demand, pure demand-side measures have little impact on real variables, indicating that a small open economy like the Federation of Bosnia and Herzegovina has only little scope for influencing macroeconomic developments with pure demand management policies. Our results confirm earlier theoretical and empirical studies showing that the labour market can best be influenced positively by reducing the tax wedge. The multipliers of income tax reductions are larger and oscillate more than the effects of the other fiscal policy measures.

## Introduction

The economic and financial crisis of 2007–2009, meanwhile known as the “Great Financial Crisis” or the “Great Recession”, the following fiscal consolidation phase, and more recently the sharp recession due to the Coronavirus pandemic, have revived the debate in academia as well as among politicians about the adequacy of active fiscal stabilisation policies. During the “Great Moderation” since the mid-1980s, stabilisation policy had been considered to be of less importance (Lucas 2003). Except for such extreme events as the lockdown measures enacted to contain the spread of the Coronavirus in 2020 and 2021, within academia opinions about the effectiveness of expansionary fiscal policy measures are sharply divided. While some authors (e.g., Taylor 2009) argue against using fiscal policy in a discretionary way, others point towards potentially large multiplier effects of tax reductions or expenditure increases (e.g., Romer and Romer 2010). Although there is a lot of evidence regarding the effects of macroeconomic policies in different countries during the Great Recession, its interpretation still diverges among macroeconomists and politicians. In particular, the role of fiscal policy is subject to ongoing controversies (see, for instance, Coenen et al. 2008, 2013; Cogan et al. 2010). Coenen et al. (2008) analyse macroeconomic effects of fiscal consolidations, while Coenen et al. (2013) and Cogan et al. (2010) analyse expansionary fiscal policies. Coenen et al. (2013) find sizeable effects of higher government consumption and investment, while they conclude that revenue-based fiscal expansions only have small effects. Coenen et al. (2008) find that both expenditure and revenue-based consolidations can have a significant impact on macroeconomic aggregates, at least in a model with an endogenous response of the equilibrium real interest rate. Cogan et al. (2010) conclude that the size of fiscal multipliers depends on the macroeconomic model used and the underlying economic theory. Compared to traditional Keynesian models, fiscal policies are much less effective in new Keynesian models.

In this paper, we aim at contributing to this debate by empirically estimating fiscal policy effects for Bosnia and Herzegovina, or to be precise for its entity named Federation of Bosnia and Herzegovina (FBIH). We are particularly interested in the question whether demand-side (Keynesian) fiscal policies aiming primarily at supporting demand can contribute to stabilising the economy or if some elements of supply-side orientation have to be added to render these policies successful. The debate between Keynesians and supply-siders was a hot topic in the 1980s in the wake of the oil price shocks and (as many macroeconomic policy debates) has not been completely settled since then. The prevailing opinion (though not a consensus) considers demand-side policies to be appropriate when combating an adverse demand-side shock but not necessarily when faced with a supply-side shock (such as stagflation). The Great Recession—as most real-world shocks—contained both demand and supply elements, but most interpretations agree that demand-side elements prevailed. Nevertheless, policies proposed by the European Commission and by the International Monetary Fund (IMF) contain calls for structural reforms to enhance growth and employment both in the short and the long term, which implies for fiscal policy to embed also supply-side measures. By contrast, many politicians and interest group representatives heavily criticize what they call the “austerity regime” of the European Commission, and advocate an expansionary fiscal policy stance in spite of already high public debt.

With the help of an econometric model, we examine the question whether the Federation of Bosnia and Herzegovina would benefit more from demand or from supply-side measures of its fiscal policy. The theoretical and empirical literature finds that in general spending multipliers are larger than tax multipliers. However, measures reducing the tax wedge not only entail demand effects by increasing disposable income, but in addition they bring about supply-side effects by increasing incentives to work or by reducing the upward pressure in wage negotiations.

## Fiscal policies in the Federation of Bosnia and Herzegovina

In this part, we provide a brief introduction to the economic and policy environment in Bosnia and Herzegovina (BIH). BIH was part of Yugoslavia, and it is an independent country since 1992. It has an extremely complicated constitutional structure, and it is composed out of two entities and one district. One of the two entities is the Federation of Bosnia and Herzegovina (FBiH), which is further composed out of ten cantons and municipalities. The entities have constitutional competencies to regulate the area of direct taxes i.e., social security contribution rates, income taxes. The central bank of BiH runs a currency board with the national currency, the Konvertible Mark (KM) pegged to the euro. In 2019 real GDP in Bosnia and Herzegovina rose by 2.8%, followed by a drop of 3.1% in 2020, mainly due to the economic consequences of the Coronavirus pandemic.

Regarding fiscal policy making, the budget preparation at the different levels is usually delayed, and due to institutional deficiencies, the functioning of the country is not efficient. Bosnia’s main challenges are low productivity, which results in low salaries, low employment rates, and high emigration. Since the country runs a currency board regime with very underdeveloped financial markets, fiscal policy is the key policy instrument for the government.

In the Federation of Bosnia and Herzegovina, fiscal policy is conducted by the government of FBiH in line with the global fiscal framework for BIH. These policies include measures of public investment, based on an annual and a 3-yerar (t, t + 1, t + 2) expenditure framework document (EFD). In addition, the issue of change in the tax wedge in FBIH is being discussed in the FBIH Parliament.^1^

## Definitions and determinants of fiscal multipliers

The Great Recession and the following fiscal consolidation revived the discussion of the effectiveness of macroeconomic stabilisation policies. In particular, the size of fiscal multipliers has been hotly debated since then, both in academia and in institutions such as central banks or the IMF. For countries that are members of a currency union such as the Euro area or that are operating under a currency board like Bosnia and Herzegovina, estimates of the effects of fiscal policy are particularly relevant since this is the only remaining macroeconomic policy instrument to deal with adverse shocks. The effectiveness of fiscal policies is usually evaluated via the size of fiscal multipliers, which are usually measured as the ratio of the change in economic output over the change in an exogenous spending or revenue item. Since the size of multipliers is likely to vary over time, various definitions of multipliers may be estimated (see, e.g., Berg 2015). The first one, the impact multiplier, measures the reaction of economic output to a change in a fiscal policy instrument in the same period t, where Y denotes output (usually GDP), INST is the policy instrument, e.g., public consumption or tax revenues, and t is the time period:

$$Impact\;multiplier = \frac{{\Delta Y_{t} }}{{\Delta INST_{t} }}$$

The multiplier at horizon *i* is defined at:

$$Multiplier\; in\;period \;t + i = \frac{{\Delta Y_{t + i} }}{{\Delta INST_{t} }}$$

Since multipliers tend first to rise, reach a peak, and decline again, it is also interesting to derive the maximum response of output to the initial fiscal shock, i.e., the peak multiplier:

$$Peak\; multiplier = \frac{{\mathop {\max }\limits_{t = 1 \ldots k} \Delta Y}}{{\Delta INST_{t} }}$$

The determination of fiscal multipliers is wide-spread in academia as well as in policy-oriented papers published by institutions like the IMF and the OECD, since they can be easily communicated and compared across countries and time periods (Čapek and Crespo Cuaresma 2020). Furthermore, the precision of the estimation of fiscal multipliers contributes significantly to the quality of GDP growth predictions (Blanchard and Leigh 2013). In the latter study, the authors find that in advanced economies stronger planned fiscal consolidation in the period after the Great Financial Crisis was associated with lower growth than expected. The likely reason for this is that fiscal multipliers were substantially higher than assumed by forecasters.

Despite their abundance, theoretical and empirical studies have not yet reached a consensus on the size of fiscal multipliers, nor on the question how to design fiscal policies when facing a severe crisis or when consolidating the budget. What has been identified, however, are the factors that determine the effectiveness of fiscal policies. These factors comprise trade openness, labour market rigidities, the size of automatic stabilisers, the exchange rate regime, the share of credit-constrained consumers, the debt level, the effectiveness of fiscal administration, the state of the economy in the business cycle, as well as the stance of monetary policy (see, e.g., Batini et al. 2014).

Fiscal policies are less effective ceteris paribus in small open economies than in larger and less open economies, but even for open economies the empirical evidence is mixed. Especially for small open economies, an internationally coordinated fiscal action might be more effective than isolated policies. Furthermore, an already high level of public debt is likely to undermine positive effects of fiscal stimuli. Hence, a clear commitment to fiscal consolidation after overcoming a crisis is required (see, e.g., Spilimbergo et al. 2009; IMF 2008). Fiscal multipliers may also vary with the position in the business cycle. Auerbach and Gorodnichenko (2013) conclude that spending multipliers tend to be larger in recessions than in expansions. Furthermore, strict fiscal consolidation measures in a recession might contribute to a deepening of the recession (Blanchard and Leigh 2013). Labour market rigidities increase fiscal multipliers if these rigidities reduce wage flexibility, since the response of output to a fiscal shock is larger if wages react only little. Lage automatic stabilisers reduce multipliers of active fiscal policies as the automatic response of taxes or spending offsets part of the initial fiscal policy action (Batini et al. 2014).

Regarding the design of fiscal policies, it is still an open issue whether taxes or public spending are more effective at stimulating output and employment (see, for example, Erceg and Lindé, 2013; Bouakez et al. 2014; Dufrénot et al. 2016). The side effects on government debt, which are a relevant problem in view of the relatively high debt to GDP ratios in many industrialised countries, may also be affected differently by revenue and expenditure side measures. Using a similar approach as in the present paper, Weyerstrass et al. (2020) find that those fiscal policy measures that entail both demand and supply-side effects are more effective at stimulating output than pure demand-side measures. Employment can be most effectively stimulated by cutting the income tax rate and the social security contribution rate, i.e., by reducing the tax wedge on labour income and positively affecting the country’s international competitiveness. Except for higher spending on research and development, all fiscal policy measures lead to higher public debt.

## A macroeconometric model of the Federation of Bosnia and Herzegovina

**Fig. 1:**
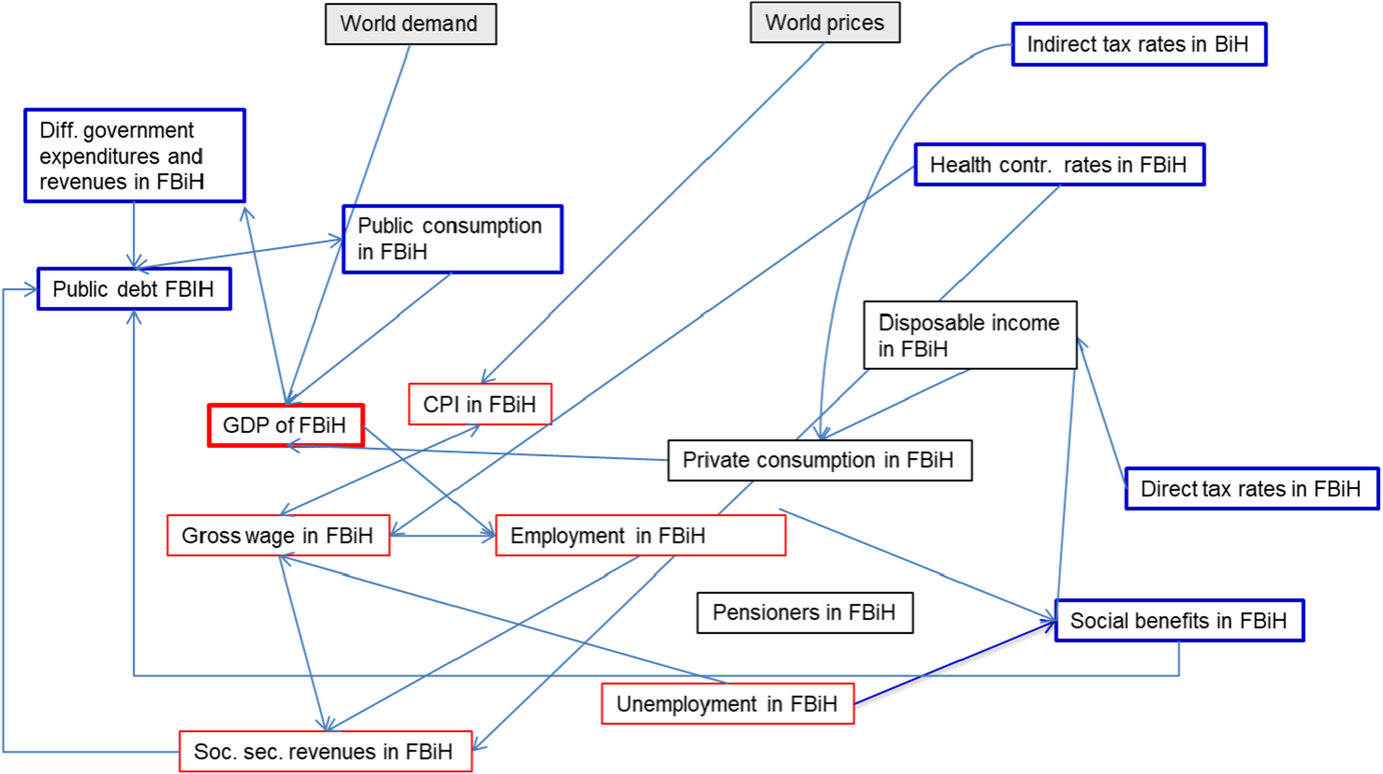
Schematic representation of the macroeconomic model of the FBiH. (*Source: author’s own visualisation)*

For the simulations, we use a macroeconometric model of the Federation of Bosnia and Herzegovina. The model was estimated with annual data for the period 2000–2018. However, for many variables the period for which data are available is much shorter. In principle, the model is comparable to a model for the entire country Bosnia and Herzegovina as described in Weyerstrass (2009). One crucial difference is that the latter model also includes a supply block determining potential GDP via a production function. Due to the lack of capital stock data on the entity level, the model for the FBiH cannot contain such a supply part. Since most time series have a unit root, i.e., they are non-stationary in levels, but stationary in first differences, almost all behavioural equations were estimated in error-correction form. The model equations are shown together with a variable list in the appendix. In the model, GDP in the FBiH economy is influenced by international demand and by domestic demand in the Federation. Domestic demand comprises consumption of private households, public consumption, and gross fixed capital formation. Private consumption is determined on the level of the Federation. It is influenced by disposable income, which in turn depends on taxes and social security contributions as well as transfers in the FBiH. Investment is also determined on the level of the Federation. It is influenced by GDP as the overall activity variable and by the real interest rate. Public consumption is determined in a Taylor type fiscal rule. In this rule, public consumption is positively linked to the past development of the fiscal balance. The notion behind this modelling is that an increase in a fiscal deficit should be counteracted in the following period by a public spending restraint, while an improvement in the fiscal balance allows the government to spend more. In the simulations, we turn this equation off and instead treat government consumption as an exogenous policy variable, the variation of which determines one of the fiscal multipliers. Exports of the FBiH are influenced by international demand, which is approximated by aggregated GDP in the Federation’s ten most important trading partners, and by the real effective exchange rate of the Bosnian currency, the Konvertible Mark (KM). Imports depend on final demand, while an influence of the exchange rate was not supported by the data. Employment, i.e., labour demand by companies, depends positively on GDP and negatively on the net wage plus the tax wedge. For policy simulations different from those described in this paper, we differentiated employment by three qualification levels. The influence of the tax wedge on labour demand varies with the qualification level. The tax wedge, i.e., the ratio between the net and the gross wage, where the gross wage is computed by summing net wage and the contributions to the various parts of social security: the health, pension, unemployment, and accident insurance. Labour supply is determined via the participation rate. It positively depends on the gross wage. In the price block, consumer prices and the GDP deflator are determined. The consumer price index (CPI) depends on the gross wage as the most important domestic cost factor, and on world prices. The latter are approximated by the HWWI index of raw material prices. The GDP deflator is explained by the CPI. In the public sector block, collections from social security contributions, value added tax (VAT) revenues, income tax revenues, other tax revenues, other revenues, social benefits, subsidies, public consumption, other expenditures, and the budget balance are determined. The revenues are determined by multiplying the relevant tax base by the respective tax or contribution rate. VAT revenues are determined in an estimated equation with total private consumption, multiplied by the normal VAT rate, as determinant. The use of a behavioural instead of a definition equation is necessary to account for tax exemptions. Social benefits, which are an important part of disposable income, are explained by health care expenditures and by pensions. Health care expenditures are determined by the revenues of the health fund. Pensions are calculated by the number of pensioners, multiplied by the average pension. As in the case of government consumption, for the determination of fiscal multipliers the equation determining social benefits is turned off, thus making social benefits exogenous in the model, which allows treating them as a policy instrument. Figure 1 shows an outline of the FBiH model.

The following FBiH-specific policy instruments are implemented in the model for the FBiH.

- Pension funds contribution rate in FBiH
- Contribution rate for Health Insurance, FBiH
- Contribution rate for Unemployment Insurance, FBiH
- Income tax rates (personal and corporate)
- Value added tax (VAT) rate
- Benefits from social security
- Public consumption in FBiH

Although our macroeconometric FBiH model is used for forecasting and policy simulations, it should be noted that the model—like every structural econometric model—may be subject to the famous Lucas critique. Lucas (1976) argued that the relations between macroeconomic aggregates in an econometric model change when the macroeconomic policy regime changes. In this case, the effects of a new policy regime cannot be predicted using an empirical model based on data from previous periods when that policy regime was not in place. As Sargent (1981) argues, the Lucas critique is partly based on the notion that the parameters of an observed policy rule should not be viewed as structural. Instead, structural parameters in Sargent’s conception are “deep parameters” such as preferences and technologies. These parameters would be invariant, even under changing policy regimes. Providing for such “deep parameters” requires a different class of macroeconomic models, namely computable general equilibrium (CGE) or dynamic stochastic general equilibrium (DSGE) models. An approach taking the Lucas critique into account in structural models like ours emerged in London School of Economics’ tradition initiated by Sargan (1964). According to this approach, economic theory guides the determination of the underlying long-run specification, while the dynamic adjustment process is derived from an analysis of the time series properties of the data series. Error correction models involving cointegrated variables combine the long-run equilibrium and the short-run adjustment mechanism.

## Simulation design

We estimate the multipliers, i.e., the effects of changes in the various fiscal policy instrument, by performing different simulations with our macroeconometric model. To this end, we first run a baseline simulation over the period 2019–2030, assuming plausible paths of the exogenous variables like world demand and international raw material prices as well as the fiscal policy instruments. Then, we implement changes to the policy instruments, one instrument at a time, and perform new simulations. We then calculate differences in important macroeconomic aggregates (nominal and real GDP, inflation, employment, unemployment rate, net exports, and the budget balance in relation to GDP) between the various policy simulations and the baseline simulation. For the baseline simulation, we assume that, due to the Coronavirus pandemic, demand from the Federation’s most important trading partners decreases by 3.5% in 2020 and rises by 3.5% in 2021 and by 3% p.a. from 2022 onwards. World raw material prices are assumed to collapse by 30% in 2020 (after having decreased by 10% in 2019), before rising by 30% in 2021 and b 5% annually from 2022 onwards. For the FBiH’s working-age population we assume a continuation of the declining trend, while the number of pensioners should continue to rise. Regarding the fiscal policy variables, we hold the tax and social security contribution rates constant at their latest observed values, varying between 2018 and 2019. For government consumption, we take the results from a first baseline simulation with government consumption being endogenously determined via a fiscal rule. This results in quite low growth rates of 1.9% in 2020, 0.3% in 2021, and 0.5% in 2022. Then the growth rate of public consumption gradually picks up to 3.3% in 2030. Similarly, the path of social benefits is firstly determined endogenously in an estimated equation. This simulation gives a negative change of social benefits by 4.3% in 2020. Afterwards, these transfers rise, with the growth rate increasing from 3.2% in 2021 to 6% in 2030. In the simulations determining the fiscal multipliers, we generally take these paths of public consumption and social benefits, only changing them in the respective policy simulations.

These assumptions and settings result in paths of the macroeconomic aggregates of the FBiH during the period 2019 to 2030, forming the basis on which to determine the fiscal multipliers. Due to the Coronavirus pandemic, the model predicts real GDP in the Federation to decline by 4.0% in 2020 and to grow by 7.3% in 2021. Afterwards, the real growth rate gradually moderates from 5.7% in 2022 to 4.7% in 2030. This brings about a gradual reduction in unemployment. The unemployment rate first rises from 38.4% in 2019 to 41.1% in 2020, but then decreases to just 19% in 2030. Of course, this favourable development is not caused by the real GDP growth alone, but also by the decline of the population in working age. Due to the Coronavirus pandemic, employment decreases by 4.7% in 2020, followed by an increase of 3.4% in 2021. Afterwards, the annual growth rate of the number of employees settles at around 3.5% until 2030. The consumer price index is forecast to decrease by 2.3% in 2020, which is due to the collapse in international raw material prices because of the Coronavirus crisis. Then, the inflation rate becomes positive in 2021, reaching 1.3% at the end of the simulation period.

For determining the macroeconomic impacts of different fiscal policies, we perform various policy experiments in which we change, one in each simulation, the fiscal policy instruments. For each instrument, we distinguish two simulations, one with permanent and one with temporary fiscal policy measures. For the permanent fiscal policy measures, we implement changes to the instruments from the year 2020 onwards, while in the scenarios of temporary measures we implement these changes in the year 2020 only. We assume an increase in government consumption and social benefits by 100 million KM each. The magnitude of the simulation experiment is taken arbitrarily but it is based on the fiscal size of these components in the FBIH budget, and it is comparable to the revenue measures i.e., the initial revenue loss is approximately 100 million KM. Also, the magnitude was specified in such a way to avoid potential distortions in the market mechanism, monetary policy reactions or large budget deficits that could lead to violations of fiscal rules. Specifically, we perform the experiments summarized in Table 1.

**Table 1:** Overview of fiscal policy experiments

	Temporary measures	Permanent measures
Spending measures	Government consumption + 100 mill. KM in 2020	Government consumption + 100 mill. KM from 2020 onwards
	Social benefits + 100 mill. KM in 2020	Social benefits + 100 mill. KM from 2020 onwards
Revenues measures	Value added tax rate − 2.5 pp in 2020	Value added tax rate − 2.5 pp from 2020 onwards
	Personal income tax rate − 5 pp in 2020	Personal income tax rate − 5 pp from 2020 onwards
	Health care contribution rate − 4 pp in 2020	Health care contribution rate − 4 pp from 2020 onwards

The policy instruments work through different channels. The spending measures entail a demand-side effect only, either directly (government consumption) or indirectly by raising disposable income of private households (social benefits). Also, reductions in the VAT rate are primarily directed towards demand. To the contrary, reductions in the income tax rate as well as in the social security contribution rate decrease the tax burden on labour income. By reducing the tax wedge between gross and net wages, they reduce the pressure on gross wages in wage negotiations. Thus, these measures decrease labour costs, thereby increasing incentives for companies to employ more workers. Regarding the scenario with the reduction in the social security contribution rate, we chose health care as the representative social security branch. In addition to health care, the social security system comprises the unemployment insurance, the pension insurance, and the accident insurance. Contributions to the social security system are in general divided between employers and employees. Hence, we implemented the reduction in the social security contribution rate by cutting both the employers’ and the employees’ rates by 2 percentage points each. Which side bears the social security contributions depends on their incidence, which in turn is determined by the relative power of trade unions and employer associations in the wage negotiations.

## Simulation results—interpretation of fiscal multipliers

**Fig. 2:**
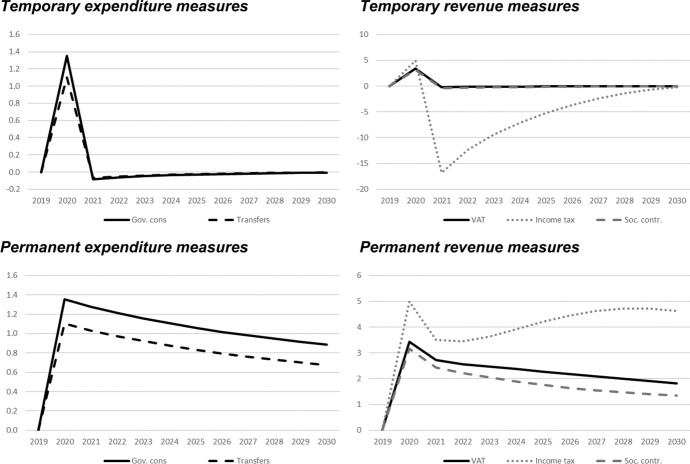
Real GDP (million 2015 KM). (*Source: authors’ own calculations and illustration)*

**Fig. 3:**
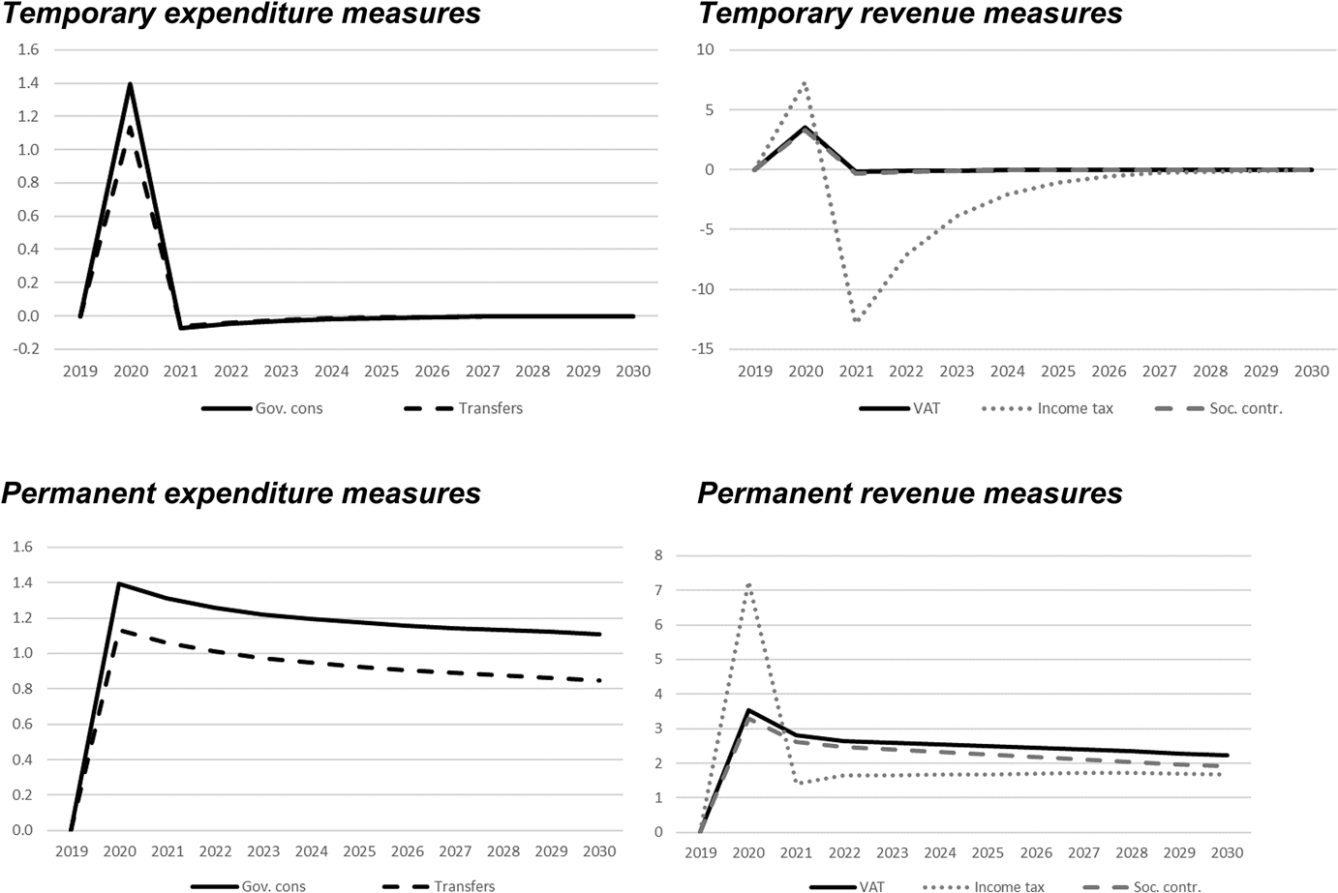
Nominal GDP (million KM). (*Source: authors’ own calculations and illustration)*

**Fig. 4:**
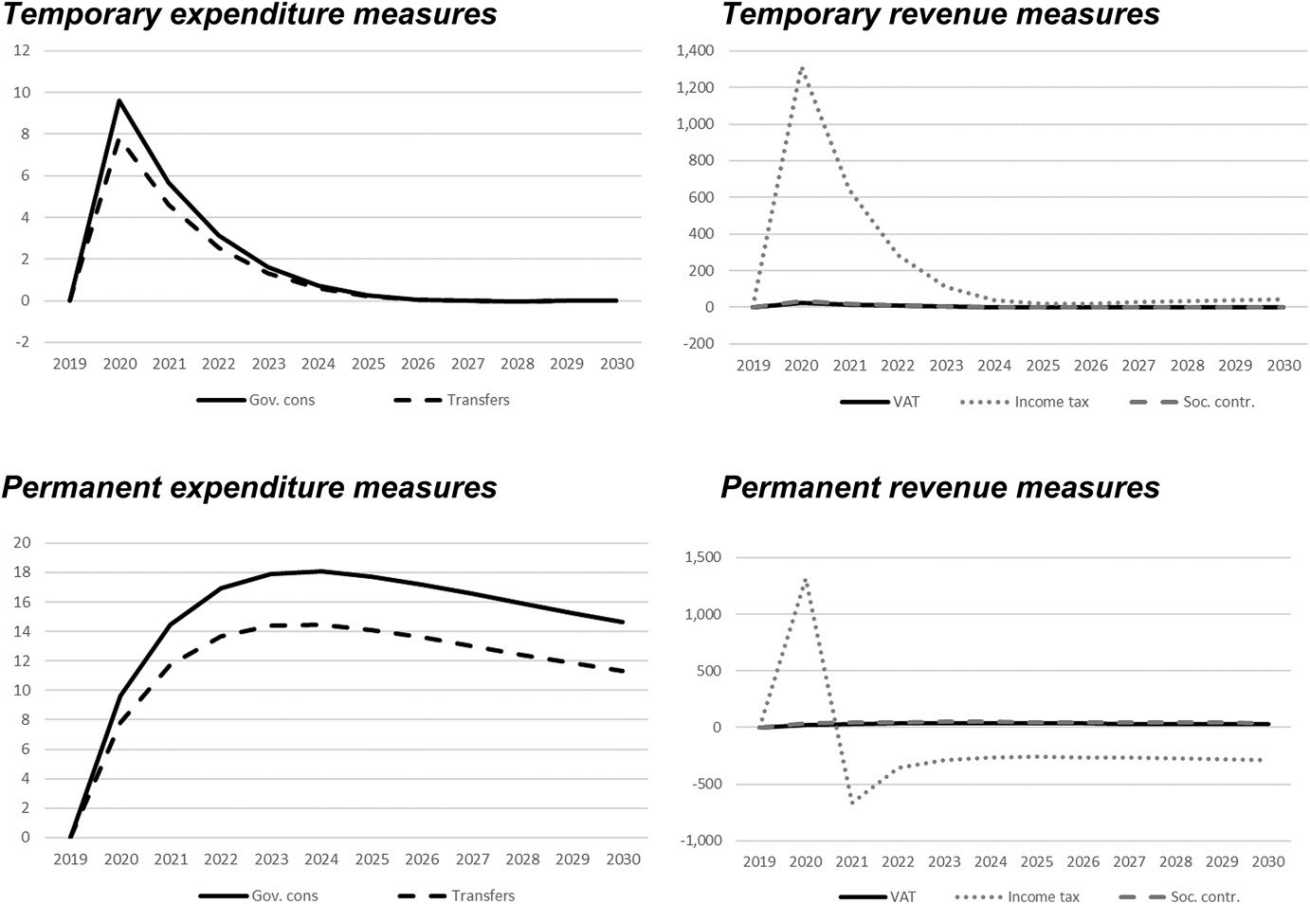
Employment (number of employees). (*Source: authors’ own calculations and illustration)*

**Fig. 5:**
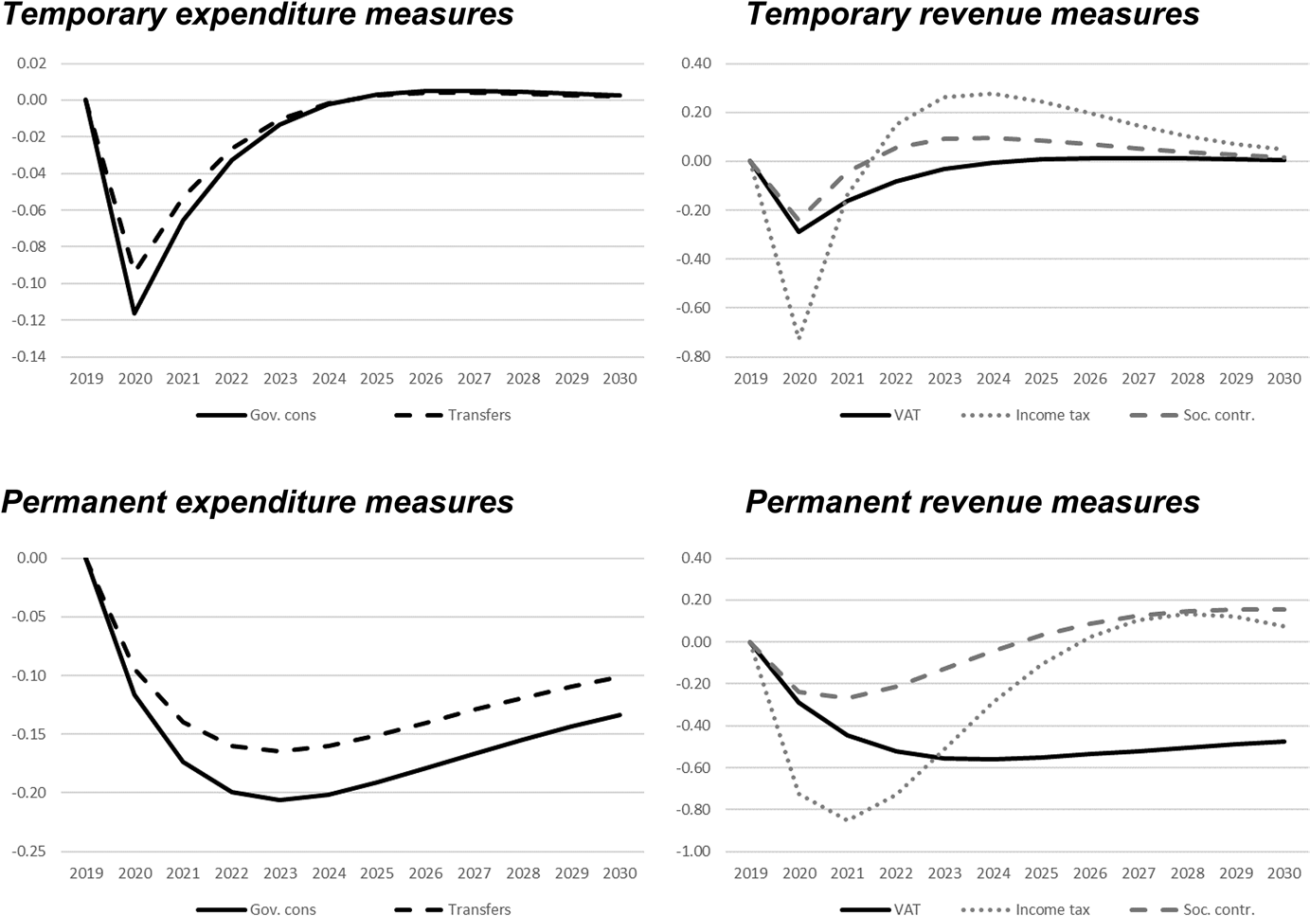
Unemployment rate (percentage points). (*Source: authors’ own calculations and illustration)*

**Fig. 6:**
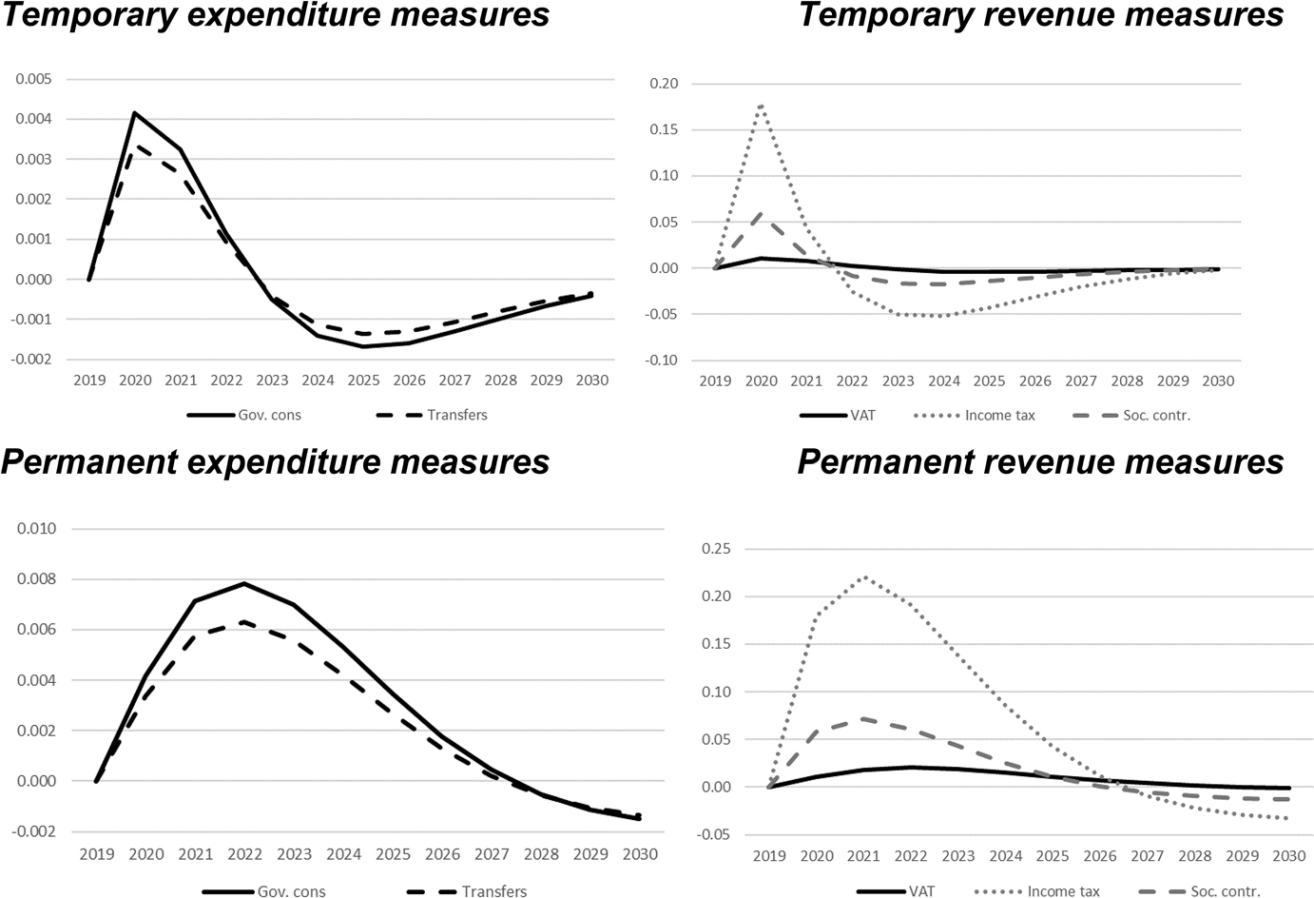
Inflation rate (percentage points). (*Source: authors’ own calculations and illustration)*

**Fig. 7:**
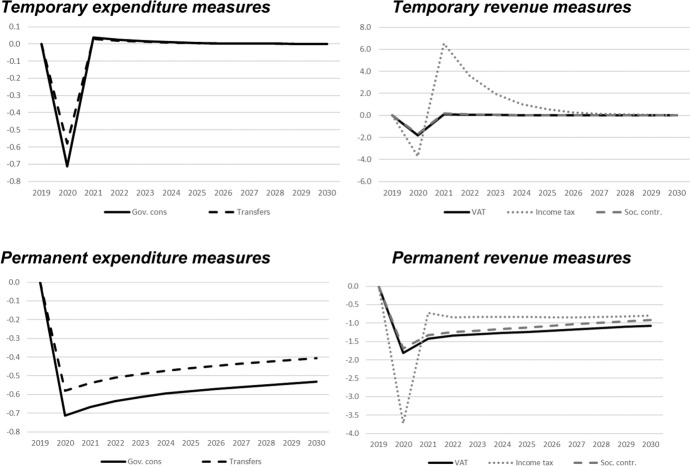
Net exports (million KM). (*Source: authors’ own calculations and illustration*)

**Fig. 8:**
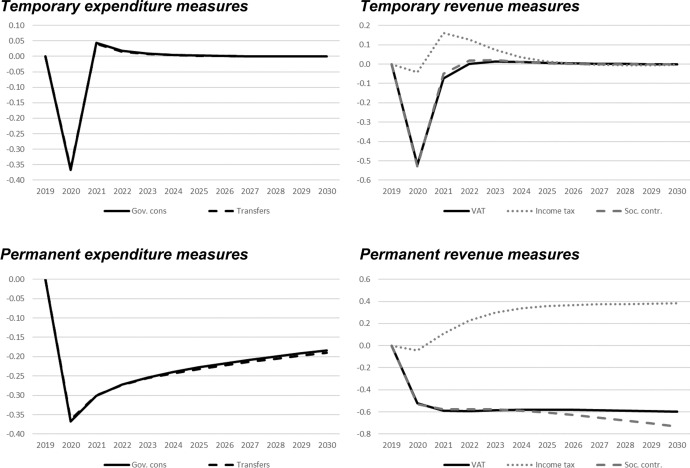
Government fiscal balance in relation to GDP (percentage points). (*Source: authors’ own calculations and illustration*)

In this section, we show the results of the previously described policy scenarios. In each of the following figures, deviations of the respective macroeconomic aggregate from the baseline simulation are depicted. Since we focus not only on nominal GDP but also on other important macroeconomic aggregates (inflation, employment, unemployment, net exports, public budget), not all results are fiscal multipliers in the strict sense. However, we normalised the results by dividing the change in the respective variables by the change in total expenditures or total revenues, respectively. Exceptions are the results for the inflation rate and for the unemployment rate, since these impacts would be extremely small in the case of the mentioned normalisation. For these two variables, the absolute deviations between the baseline and the alternative scenarios as described in Table 1 are shown in the figures. The effects are shown in Figs. 2 , 3, 4, 5, 6, 7 and 8. In addition, for the temporary measures, Table 2 summarises the impact multipliers, the multipliers after 5 years, and the maximum effects.

**Table 2:** Summary of multipliers (*Source: authors’ own calculations*)

	Gov. cons	Transfers	VAT	Income tax	Contributions
*On impact*					
Nominal GDP	1.4	1.1	3.5	7.3	3.3
Real GDP	1.4	1.1	3.4	5.0	3.2
Employment	9.6	7.8	24.3	1316.5	33.9
Inflation rate	0.004	0.003	0.010	0.179	0.058
Budget balance	− 0.4	− 0.4	− 0.5	0.0	− 0.5
Net exports	− 0.7	− 0.6	− 1.8	− 3.7	− 1.7
*After 5 years*					
Nominal GDP	0.0	0.0	0.0	− 1.1	0.0
Real GDP	0.0	0.0	− 0.1	− 5.2	− 0.1
Employment	0.3	0.2	0.7	18.1	0.9
Inflation rate	− 0.002	− 0.001	− 0.004	− 0.043	− 0.014
Budget balance	0.0	0.0	0.0	0.0	0.0
Net exports	0.0	0.0	0.0	0.5	0.0
*Maximum (absolute)*					
Nominal GDP	1.4	1.1	3.5	7.3	3.3
Real GDP	1.4	1.1	3.4	5.0	3.2
Employment	9.6	7.8	24.3	1316.5	33.9
Inflation rate	0.004	0.003	0.010	0.179	0.058
Budget balance	− 0.4	− 0.4	− 0.5	0.0	− 0.5

As one would expect, the GDP effects of increases of public consumption are a bit larger than those of increases in social benefits of the same magnitude (Figs. 2, 3). Regarding the tax measures, reductions of the income tax rate have the smallest effect, and the effects become negative over time in the case of a temporary tax reduction. Although our model focuses on the demand-side, it generates typical supply-side effects of some fiscal policies. In particular, employment can be very effectively increased, and hence unemployment reduced, by measures that reduce the tax wedge or labour costs (Figs. 4, 5). Interestingly, these positive labour market effects are rather short-lived. This result is in line with Weyerstrass et al. (2020) for Slovenia.

The inflationary impacts of the fiscal policy measures are small (Fig. 6). Interestingly, the inflation effects of the temporary measures even become negative over time, in particular regarding the temporary demand-side effects. A negative effect means that inflation with the implementation of the measure is lower than inflation in the baseline simulation. All fiscal policy measures, except for the income tax reduction in some years, decrease net exports (Fig. 7). This is to be expected since the measure (at least temporarily) raise domestic demand and hence imports. Furthermore, the (although only slightly) higher inflation leads to a real appreciation of the Bosnian currency which is also detrimental to net exports. Finally, of course all expansionary fiscal policy measures deteriorate public finances (Fig. 8). Striking is the profile of the reaction of all macroeconomic variables to changes in the income tax rate. This fiscal policy measure entails much larger effects than the other measures in the first years after implementation, and the multipliers oscillate over time. In the model, the reaction of employment to changes in the personal income tax rate is substantial, making this instrument highly effective in influencing the macroeconomy.

In line with the definition of multipliers is Sect. 3, Table 2 summarises the impact multipliers, the multipliers after 5 years, and the maximum multipliers for the temporary policy measures. For this analysis, we focus on the temporary measures, since regarding the sustainability of public finances, temporary measures are more appropriate than permanent expansionary measures. This is also in line with recommendations of institutions like the IMF or the OECD that fiscal stimuli should by timely, targeted, and temporary. The impact multiplier of the income tax reduction is the largest, followed by the other tax measures. The applies to GDP and employment. Also, the maximum multipliers of the tax reduction are larger than the effects of the other measures. However, after 5 years, most multipliers are zero, i.e., the macroeconomic variables have returned to their baseline values, but the multipliers of the tax reduction are negative after 5 years.

## Conclusions

The Great Recession of 2007–2009, and more recently the sharp recession due to the Coronavirus pandemic, have revived the debate in academia as well as among politicians about the adequacy of active fiscal stabilisation policies. We use a macroeconometric model for the Federation of Bosnia and Herzegovina to explore the macroeconomic impacts of different expenditure and revenue based fiscal policy measures. Our results show that those measures that reduce the tax wedge on labour income are particularly effective in stimulating employment. Due to the high elasticity of imports with respect to demand, pure demand-side effects on real variables are small, showing that a small open economy like the Federation of Bosnia and Herzegovina pure demand-side policy measures have only limited scope for influencing macroeconomic developments.

It would be premature to suggest strong and precise recommendations for the current macroeconomic situation of the Bosnian or the Federation’s economy based on just one model specification. It should be noted, however, that in a recent study with a New Keynesian DSGE model, Sims and Wolff (2018) show that countercyclical fiscal policies are more successful when using productive public investment instead of public consumption. Since due to lack of data our model does not incorporate potential GDP, we cannot explore these effects in our model. Our results do confirm earlier theoretical and empirical studies showing that the labour market can best be influenced positively by reducing the tax wedge.

## Appendix: The macroeconometric model of the Federation of Bosnia and Herzegovina

This appendix provides a list of all behavioural equations and identities of the model used for the simulation. A list of the variables is provided below the equations. The equations can be grouped into different blocks:

- GDP and its components: nominal and real GDP, gross fixed capital formation, private consumption, public consumption, exports, imports. Public consumption can also be grouped into the public sector part of the model (see below).
- Prices and wages: gross and net wage, consumer price index (CPI), GDP deflator.
- Labour market: employment, total as well as divided into three qualification levels (low, medium, high), unemployment, number of pensioners.
- Public sector: collections from social security contributions, VAT revenues, income tax revenues, other tax revenues, other revenues, social benefits, subsidies, (public consumption), other expenditures, budget balance.

### Private consumption

Consumption of private households (HCP) depends on disposable income (YDP).

**Table:**

Variable	Coefficient	Std. error	t-Statistic	Prob.
*Dependent variable: LOG(HCP)*				
C	4.807561	0.421261	11.41232	0.0000
LOG(YDP)	0.530457	0.047133	11.25453	0.0000
R-squared	0.947630	Mean dependent var		9.547380
Adjusted R-squared	0.940149	S.D. dependent var		0.119636
S.E. of regression	0.029268	Akaike info criterion		− 4.031493
Sum squared resid	0.005996	Schwarz criterion		− 3.987666
Log likelihood	20.14172	Hannan–Quinn criter		− 4.126073
F-statistic	126.6646	Durbin–Watson stat		1.429864
Prob(F-statistic)	0.000010			

### Gross fixed capital formation

Investment, or gross fixed capital formation (GFCFP) is influenced by output, approximated by the most comprehensive measure of output, i.e. GDP (GDPP), and by the real interest rate, i.e. the nominal interest rate (INTRATE) minus annual inflation (@pcy(CPI)). In addition a dummy variable for 2007 was included.

**Table:**

Variable	Coefficient	Std. error	t-Statistic	Prob.
Dependent variable: LOG(GFCFP)				
C	0.895740	1.753502	0.510829	0.6233
LOG(GDPP)	0.749348	0.180658	4.147882	0.0032
INTRATE-@PCY(CPI)	− 0.026512	0.012753	− 2.078928	0.0712
D2007	0.263721	0.123927	2.128042	0.0660
R-squared	0.851367	Mean dependent var		7.886264
Adjusted R-squared	0.795629	S.D. dependent var		0.261635
S.E. of regression	0.118278	Akaike info criterion		− 1.170352
Sum squared resid	0.111918	Schwarz criterion		− 1.008716
Log likelihood	11.02211	Hannan–Quinn criter		− 1.230195
F-statistic	15.27456	Durbin–Watson stat		1.845390
Prob(F-statistic)	0.001127			

### Exports of goods and services

Exports of goods and services (XP) depend on the lagged dependent variable, i.e. exports in the previous year, and on demand in Bosnia and Herzegovina’s most important trading partners (WORLDDEMAND). World demand is defined as the sum of GDP in Germany, Austria, Italy, Slovenia, Croatia, Serbia, Montenegro, and USA, all in current prices and in euro. Furthermore, a dummy variable for 2009 had to be included.

**Table:**

Variable	Coefficient	Std. error	t-Statistic	Prob.
Dependent variable: LOG(XP)				
LOG(XP(−1))	0.817728	0.081787	9.998306	0.0000
LOG(WORLDDEMAND)	0.098763	0.040324	2.449259	0.0442
D2009	− 0.307902	0.092379	− 3.333040	0.0125
R-squared	0.946085	Mean dependent var		8.291203
Adjusted R-squared	0.930681	S.D. dependent var		0.320987
S.E. of regression	0.084511	Akaike info criterion		− 1.860543
Sum squared resid	0.049995	Schwarz criterion		− 1.769768
Log likelihood	12.30272	Hannan–Quinn criter		− 1.960124
Durbin–Watson stat	1.682920			

### Imports of goods and services

Imports of goods and services (MP) are influenced by domestic demand, approximated by GDP. In addition, dummies for 2009 and 2010 had to be included.

**Table:**

Variable	Coefficient	Std. error	t-Statistic	Prob.
Dependent variable: LOG(MP)				
C	− 0.280964	1.256439	− 0.223619	0.8287
LOG(GDPP)	0.982717	0.132119	7.438113	0.0001
D2009 + D2010	− 0.163865	0.067246	− 2.436784	0.0408
R-squared	0.873713	Mean dependent var		9.060643
Adjusted R-squared	0.842141	S.D. dependent var		0.205940
S.E. of regression	0.081823	Akaike info criterion		− 1.941516
Sum squared resid	0.053560	Schwarz criterion		− 1.832999
Log likelihood	13.67834	Hannan–Quinn criter		− 2.009920
F-statistic	27.67380	Durbin–Watson stat		1.164306
Prob(F-statistic)	0.000254			

### Public consumption (fiscal statistics)

In a Taylor type fiscal rule, government consumption (GOVCONS) depends on the lagged budget balance (BALGDP). An improvement in the budget balance allows the government to spend more in the following year, while a deterioration of the fiscal balance has to be counteracted by a spending restraint. Furthermore, lagged public consumption influences current public consumption since public expenditures cannot be adjusted quickly. Dummies for 2007 and 2008 are also included.

**Table:**

Variable	Coefficient	Std. error	t-Statistic	Prob.
Dependent variable: LOG(GOVCONS)				
C	0.264805	0.561075	0.471961	0.6568
LOG(GOVCONS (− 1))	0.968948	0.072065	13.44541	0.0000
BALGDP(− 1)	0.011561	0.006732	1.717418	0.1465
D2007 + D2008	0.081183	0.019000	4.272757	0.0079
R-squared	0.992416	Mean dependent var		7.814132
Adjusted R-squared	0.987866	S.D. dependent var		0.147287
S.E. of regression	0.016224	Akaike info criterion		− 5.103512
Sum squared resid	0.001316	Schwarz criterion		− 5.015857
Log likelihood	26.96581	Hannan–Quinn criter		− 5.292672
F-statistic	218.1025	Durbin–Watson stat		3.006938
Prob(F-statistic)	0.000010			

### Nominal GDP

Nominal GDP could theoretically be determined by adding up the expenditure components. However, data on the level of the entities is less accurate than data for the state level. In particular, it is difficult to measure exports and imports for the entities. Furthermore, capital formation does not include changes in inventories. In addition, public consumption refers to fiscal statistics, not the national accounts, and there are some discrepancies between these two concepts. Hence, GDP is determined in a behavioural equation by the expenditure components (HCP, GFCFP, XP, MP). A dummy for 2013 is also considered.

**Table:**

Variable	Coefficient	Std. error	t-Statistic	Prob.
Dependent variable: LOG(GDPP)				
C	0.777577	0.322087	2.414181	0.0465
LOG(HCP + GOVCONS + GFCFP + XP-MP)	0.922731	0.033861	27.25017	0.0000
D2013	− 0.088304	0.021967	− 4.019933	0.0051
R-squared	0.991646	Mean dependent var		9.571284
Adjusted R-squared	0.989259	S.D. dependent var		0.179087
S.E. of regression	0.018560	Akaike info criterion		− 4.892261
Sum squared resid	0.002411	Schwarz criterion		− 4.801486
Log likelihood	27.46131	Hannan–Quinn criter		− 4.991842
F-statistic	415.4583	Durbin–Watson stat		2.207025
Prob(F-statistic)	0.000000			

### Gross wage rate

Wages depend positively on prices and negatively on the unemployment rate. The elasticity of gross nominal wages (GWAGE) with respect to consumer prices (CPI) have been restricted to 1 so as to ensure that in the long run real wages do not decline. The negative influence of the unemployment rate follows the notion that an improvement in the labour market situation gives employees or trade unions, respectively, more power in wage negotiations, while an increase of the unemployment rate negatively affects the employees’ negotiation power.

**Table:**

Variable	Coefficient	Std. error	t-Statistic	Prob.
Dependent variable: LOG(GWAGE/CPI(-1))				
C	2.243427	0.042439	52.86252	0.0000
D(UR)	− 0.062824	0.030714	− 2.045450	0.0655
R-squared	0.275547	Mean dependent var		2.202585
Adjusted R-squared	0.209687	S.D. dependent var		0.151881
S.E. of regression	0.135021	Akaike info criterion		− 1.026129
Sum squared resid	0.200539	Schwarz criterion		− 0.939213
Log likelihood	8.669835	Hannan–Quinn criter		− 1.043994
F-statistic	4.183867	Durbin–Watson stat		0.387862
Prob(F-statistic)	0.065487			

### Net wage rate

Net wages (NWAGE) are gross wages (GWAGE) minus social security contributions. Hence, contribution rates to the pension insurance (CRPENSION), health insurance (CRHEALTH) and to the unemployment insurance (CRUN), both for employees (_EE) and employers (_ER) have to be taken into account.

**Table:**

Variable	Coefficient	Std. error	t-Statistic	Prob.
Dependent variable: NWAGE				
C	50.68668	10.18342	4.977373	0.0006
(GWAGE-GWAGE*(CRPENSION_ER + CRHEALTH_ER + CRUN_ER)/100)*(1-(CRPENSION_EE + CRHEALTH_EE + CRUN_EE + PERSINCTAXRATE)/100)	1.171137	0.018475	63.38944	0.0000
R-squared	0.997518	Mean dependent var		682.9167
Adjusted R-squared	0.997269	S.D. dependent var		136.2828
S.E. of regression	7.121650	Akaike info criterion		6.915168
Sum squared resid	507.1790	Schwarz criterion		6.995986
Log likelihood	− 39.49101	Hannan–Quinn criter		6.885246
F-statistic	4018.221	Durbin–Watson stat		1.047381
Prob(F-statistic)	0.000000			

### Consumer price index (CPI)

Consumer prices (CPI) are influenced by domestic costs, approximated by labour costs, which are in turn represented by the average gross wage (GWAGE), and by international raw material prices (HWWI).

**Table:**

Variable	Coefficient	Std. error	t-Statistic	Prob.
Dependent variable: LOG(CPI)				
C	2.568726	0.212288	12.10019	0.0000
LOG(GWAGE)	0.242881	0.048253	5.033457	0.0004
LOG(HWWI)	0.104222	0.033125	3.146317	0.0093
R-squared	0.965520	Mean dependent var		4.683053
Adjusted R-squared	0.959251	S.D. dependent var		0.103907
S.E. of regression	0.020975	Akaike info criterion		− 4.703546
Sum squared resid	0.004840	Schwarz criterion		− 4.566605
Log likelihood	35.92482	Hannan–Quinn criter		− 4.716222
F-statistic	154.0128	Durbin–Watson stat		0.627758
Prob(F-statistic)	0.000000			

### GDP deflator

The GDP deflator (PGDP) depends on consumer prices (CPI).

**Table:**

Variable	Coefficient	Std. error	t-Statistic	Prob.
Dependent variable: LOG(PGDP)				
C	− 2.001681	0.466931	− 4.286886	0.0011
LOG(CPI)	1.429032	0.099684	14.33565	0.0000
R-squared	0.944830	Mean dependent var		4.690552
Adjusted R-squared	0.940233	S.D. dependent var		0.152761
S.E. of regression	0.037346	Akaike info criterion		− 3.605625
Sum squared resid	0.016737	Schwarz criterion		− 3.514331
Log likelihood	27.23937	Hannan–Quinn criter		− 3.614075
F-statistic	205.5109	Durbin–Watson stat		0.463691
Prob(F-statistic)	0.000000			

### Total employment

Employment depends (EMP) positively on GDP (GDPP) and negatively on the gross wage (GWAGE) as well as the relation between social security contribution rates in the Federation and in the Republika Srpska (RS). The latter allows the analysis of the impact of changes in social security contribution rates in the Federation relative to those in the RS. Furthermore, lagged employment plays a role since employment is typically adjusted smoothly.

**Table:**

Variable	Coefficient	Std. error	t-Statistic	Prob.
Dependent variable: LOG(EMP)				
C	4.637752	2.491784	1.861218	0.0997
LOG(EMP(−1))	0.530938	0.194070	2.735815	0.0256
LOG(GDPP)	0.162294	0.053597	3.028014	0.0164
LOG(NWAGE*(CRPENSION_ER + CRHEALTH_EE + CRUN_EE)/(CRPENSION_RS + CRHEALTH_RS + CRUN_RS + CRCHILD_RS))	− 0.018707	0.054792	− 0.341413	0.7416
R-squared	0.959081	Mean dependent var		12.93492
Adjusted R-squared	0.943736	S.D. dependent var		0.057304
S.E. of regression	0.013593	Akaike info criterion		− 5.497379
Sum squared resid	0.001478	Schwarz criterion		− 5.335744
Log likelihood	36.98428	Hannan–Quinn criter		− 5.557223
F-statistic	62.50235	Durbin–Watson stat		1.533657
Prob(F-statistic)	0.000007			

### Employment—low qualification

Also the share of persons with low qualification in total employment (EMP_LOW/EMP) depends negatively on the relation between social security contribution rates in the Federation and in the RS. Dummies for 2009 and 2010 had also to be included.

**Table:**

Variable	Coefficient	Std. error	z-Statistic	Prob.
Dependent variable: EMP_LOW/EMP				
Method: ML—censored normal (TOBIT) (quadratic hill climbing)				
Left censoring (value) series: 0				
Right censoring (value) series: 1				
Convergence achieved after 3 iterations				
Covariance matrix computed using second derivatives				
C	0.787698	0.053252	14.79194	0.0000
LOG(NWAGE*((1 + CRPENSION_ER + CRHEALTH_EE + CRUN_EE)/(1 + CRPENSION_RS + CRHEALTH_RS + CRUN_RS + CRCHILD_RS)))	− 0.108796	0.009012	− 12.07232	0.0000
D2009-D2010	0.004203	0.006970	0.603031	0.5465

**Table:**

Error distribution				
SCALE:C(4)	0.009862	0.002011	4.905111	0.0000
Mean dependent var	0.145743	S.D. dependent var		0.037390
S.E. of regression	0.012079	Akaike info criterion		− 5.733562
Sum squared resid	0.001167	Schwarz criterion		− 5.571927
Log likelihood	38.40137	Hannan–Quinn criter		− 5.793405
Avg. log likelihood	3.200114			
Left censored obs	0	Right censored obs		0
Uncensored obs	12	Total obs		12

### Employment—medium qualification

The share of persons with medium qualification in total employment (EMP_MED/EMP) depends positively on the relation between social security contribution rates in the Federation and in the RS. The notion is that non-wage labour costs are particularly detrimental to employment with low qualification. A dummy for 2008 had also to be included.

**Table:**

Variable	Coefficient	Std. error	z-Statistic	Prob.
Dependent variable: EMP_MED/EMP				
Method: ML—censored normal (TOBIT) (quadratic hill climbing)				
Left censoring (value) series: 0				
Right censoring (value) series: 1				
Convergence achieved after 3 iterations				
Covariance matrix computed using second derivatives				
C	0.385726	0.014721	26.20196	0.0000
LOG(NWAGE*((1 + CRPENSION_ER + CRHEALTH_EE + CRUN_EE)/(1 + CRPENSION_RS + CRHEALTH_RS + CRUN_RS + CRCHILD_RS)))	0.041282	0.002491	16.57118	0.0000
D2008	0.018729	0.002846	6.580663	0.0000

**Table:**

Error distribution				
SCALE:C(4)	0.002726	0.000556	4.905111	0.0000
Mean dependent var	0.630870	S.D. dependent var		0.014943
S.E. of regression	0.003339	Akaike info criterion		− 8.305095
Sum squared resid	8.92E-05	Schwarz criterion		− 8.143459
Log likelihood	53.83057	Hannan–Quinn criter		− 8.364938
Avg. log likelihood	4.485881			
Left censored obs	0	Right censored obs		0
Uncensored obs	12	Total obs		12

Employment with high qualification is calculated as the residual of total employment minus employment with low and medium qualification.

### Employment (LFS)

Employment according to the labour force survey (EMPLFS) is determined by registered employment (EMP).

**Table:**

Variable	Coefficient	Std. error	t-Statistic	Prob.
Dependent variable: LOG(EMPLFS)				
LOG(EMP)	1.011986	0.001468	689.1891	0.0000
R-squared	− 3.009905	Mean dependent var		13.12154
Adjusted R-squared	− 3.009905	S.D. dependent var		0.026892
S.E. of regression	0.053850	Akaike info criterion		− 2.888744
Sum squared resid	0.020299	Schwarz criterion		− 2.878814
Log likelihood	12.55498	Hannan–Quinn criter		− 2.955719
Durbin–Watson stat	0.628575			

### Unemployment (LFS)

Unemployment according to the labour force survey (UNLFS) is determined by registered unemployment (UN).

**Table:**

Variable	Coefficient	Std. error	t-Statistic	Prob.
Dependent variable: LOG(UNLFS)				
LOG(UN)	0.953265	0.002983	319.5984	0.0000
R-squared	0.066548	Mean dependent var		12.20741
Adjusted R-squared	0.066548	S.D. dependent var		0.111820
S.E. of regression	0.108035	Akaike info criterion		− 1.496251
Sum squared resid	0.081701	Schwarz criterion		− 1.486321
Log likelihood	6.985005	Hannan–Quinn criter		− 1.563226
Durbin–Watson stat	0.830862			

### Number of pensioners

The number of pensioners (PENSIONERS) depends on the population aged 65 and more (POP_6569 + POP_70PLUS).

**Table:**

Variable	Coefficient	Std. error	t-Statistic	Prob.
Dependent variable: LOG(PENSIONERS)				
LOG(POP_6569 + POP_70PLUS)	1.007638	0.000473	2131.113	0.0000
R-squared	0.706552	Mean dependent var		12.84468
Adjusted R-squared	0.706552	S.D. dependent var		0.022253
S.E. of regression	0.012054	Akaike info criterion		− 5.786449
Sum squared resid	0.000436	Schwarz criterion		− 5.939875
Log likelihood	12.57290	Hannan–Quinn criter		− 6.123132
Durbin–Watson stat	1.224930			

### Contributions to unemployment fund

**Table:**

Variable	Coefficient	Std. error	t-Statistic
Dependent variable: LOG(CONTUN)			
C	− 0.980002	0.159446	− 6.146309
LOG(UNCONTR)	1.008628	0.028942	34.84948
@TREND*(@YEAR < (2009))*@TREND*(@YEAR > (2003))	0.003501	0.000284	12.31533
R-squared	0.992957	Mean dependent var	
Adjusted R-squared	0.991392	S.D. dependent var	
S.E. of regression	0.021176	Akaike info criterion	
Sum squared resid	0.004036	Schwarz criterion	
Log likelihood	30.95736	Hannan–Quinn criter	
F-statistic	634.4150	Durbin–Watson stat	
Prob(F-statistic)	0.000000		

### Contributions to health fund

**Table:**

Variable	Coefficient	Std. error	t-Statistic	Prob.
Dependent variable: LOG(CONTHEALTH)				
C	− 3.357378	0.433811	− 7.739257	0.0000
LOG(HEALTHCONTR)	1.321307	0.057128	23.12888	0.0000
R-squared	0.981650	Mean dependent var		6.672228
Adjusted R-squared	0.979814	S.D. dependent var		0.297301
S.E. of regression	0.042239	Akaike info criterion		− 3.339920
Sum squared resid	0.017842	Schwarz criterion		− 3.259102
Log likelihood	22.03952	Hannan–Quinn criter		− 3.369842
F-statistic	534.9449	Durbin–Watson stat		1.323806
Prob(F-statistic)	0.000000			

### Total revenues of health fund

**Table:**

Variable	Coefficient	Std. error	t-Statistic	Prob.
Dependent variable: LOG(HEALTHREV)				
C	− 2.704082	0.355787	− 7.600285	0.0000
LOG(HEALTHCONTR)	1.285030	0.046665	27.53738	0.0000
R-squared	0.988271	Mean dependent var		7.090148
Adjusted R-squared	0.986967	S.D. dependent var		0.264550
S.E. of regression	0.030201	Akaike info criterion		− 3.998913
Sum squared resid	0.008209	Schwarz criterion		− 3.926568
Log likelihood	23.99402	Hannan–Quinn criter		− 4.044516
F-statistic	758.3072	Durbin–Watson stat		1.310848
Prob(F-statistic)	0.000000			

### Contributions to pension fund.

**Table:**

Variable	Coefficient	Std. error	t-Statistic	Prob.
Dependent variable: LOG(CONTPENSION)				
C	− 4.200378	0.260153	− 16.14578	0.0000
LOG(PENSIONCONTR)	1.410508	0.032312	43.65213	0.0000
D2003	− 0.058224	0.021966	− 2.650636	0.0292
@TREND*(@YEAR < (2009))	0.014512	0.002212	6.560018	0.0002
R-squared	0.997102	Mean dependent var		7.012277
Adjusted R-squared	0.996015	S.D. dependent var		0.303494
S.E. of regression	0.019159	Akaike info criterion		− 4.810937
Sum squared resid	0.002936	Schwarz criterion		− 4.649302
Log likelihood	32.86562	Hannan–Quinn criter		− 4.870781
F-statistic	917.4605	Durbin–Watson stat		1.888461
Prob(F-statistic)	0.000000			

### Personal income tax revenues

**Table:**

Variable	Coefficient	Std. error	t-Statistic
Dependent variable: LOG(TAXINCPERS)			
LOG(EMP*GWAGE*12*PERSINCTAXRATE/100000000)	0.856478	0.005457	156.9483
R-squared	0.757002	Mean dependent var	
Adjusted R-squared	0.757002	S.D. dependent var	
S.E. of regression	0.117488	Akaike info criterion	
Sum squared resid	0.151837	Schwarz criterion	
Log likelihood	9.191863	Hannan–Quinn criter	
Durbin–Watson stat	1.444277		

### Corporate income tax revenues

**Table:**

Variable	Coefficient	Std. error	t-Statistic	Prob.
Dependent variable: LOG(TAXINCCORP)				
C	− 15.70354	1.906058	− 8.238751	0.0000
LOG(GDPP)	2.141369	0.200830	10.66259	0.0000
D2008	− 0.521365	0.157246	− 3.315595	0.0090
R-squared	0.927028	Mean dependent var		4.604322
Adjusted R-squared	0.910812	S.D. dependent var		0.489654
S.E. of regression	0.146232	Akaike info criterion		− 0.794926
Sum squared resid	0.192454	Schwarz criterion		− 0.673700
Log likelihood	7.769558	Hannan–Quinn criter		− 0.839809
F-statistic	57.16760	Durbin–Watson stat		1.303448
Prob(F-statistic)	0.000008			

### VAT revenues

**Table:**

Variable	Coefficient	Std. error	t-Statistic	Prob.
Dependent variable: LOG(VAT)				
C	− 10.13412	2.687393	− 3.770985	0.0070
LOG(VATRATE*HCP)	1.421513	0.217056	6.549065	0.0003
R-squared	0.859692	Mean dependent var		7.465060
Adjusted R-squared	0.839648	S.D. dependent var		0.183417
S.E. of regression	0.073448	Akaike info criterion		− 2.191361
Sum squared resid	0.037762	Schwarz criterion		− 2.147533
Log likelihood	11.86112	Hannan–Quinn criter		− 2.285941
F-statistic	42.89025	Durbin–Watson stat		1.243821
Prob(F-statistic)	0.000319			

### Other tax revenues

**Table:**

Variable	Coefficient	Std. error	t-Statistic	Prob.
Dependent variable: DLOG(TAXOTHER)				
DLOG(GDPP)	0.764458	0.707969	1.079791	0.3083
D2009	− 1.840070	0.169005	− 10.88764	0.0000
R-squared	0.928194	Mean dependent var		− 0.135750
Adjusted R-squared	0.920216	S.D. dependent var		0.595055
S.E. of regression	0.168080	Akaike info criterion		− 0.565789
Sum squared resid	0.254258	Schwarz criterion		− 0.493444
Log likelihood	5.111838	Hannan–Quinn criter		− 0.611392
Durbin–Watson stat	1.803165			

#### Other revenues

**Table:**

Variable	Coefficient	Std. error	t-Statistic	Prob.
Dependent variable: REVOTHER				
GDPP	0.043380	0.005610	7.732457	0.0000
D2006 + D2007 + D2008	− 657.2232	157.0646	− 4.184414	0.0019
R-squared	0.726969	Mean dependent var		390.4639
Adjusted R-squared	0.699666	S.D. dependent var		427.9622
S.E. of regression	234.5349	Akaike info criterion		13.90410
Sum squared resid	550,066.3	Schwarz criterion		13.98492
Log likelihood	− 81.42459	Hannan–Quinn criter		13.87418
Durbin–Watson stat	0.360101			

#### Total revenues

**Table:**

Variable	Coefficient	Std. error	t-Statistic	Prob.
Dependent variable: REVTOTAL				
C	− 275.2093	209.4696	− 1.313839	0.2303
REVGROSS	0.964245	0.035826	26.91430	0.0000
D2006	616.9104	101.1238	6.100548	0.0005
R-squared	0.991093	Mean dependent var		5245.093
Adjusted R-squared	0.988548	S.D. dependent var		791.7799
S.E. of regression	84.73134	Akaike info criterion		11.96017
Sum squared resid	50,255.80	Schwarz criterion		12.05095
Log likelihood	− 56.80087	Hannan–Quinn criter		11.86059
F-statistic	389.4464	Durbin–Watson stat		1.458516
Prob(F-statistic)	0.000000			

#### Health expenditures

**Table:**

Variable	Coefficient	Std. error	t-Statistic	Prob.
Dependent variable: LOG(HEALTHEXP)				
C	− 0.305204	0.047588	− 6.413413	0.0004
LOG(HEALTHREV)	1.049322	0.006713	156.3105	0.0000
D2006 + D2007	− 0.027314	0.004357	− 6.268826	0.0004
D2008 + D2009	0.030045	0.004410	6.813237	0.0003
R-squared	0.999749	Mean dependent var		7.135144
Adjusted R-squared	0.999641	S.D. dependent var		0.283467
S.E. of regression	0.005369	Akaike info criterion		− 7.341182
Sum squared resid	0.000202	Schwarz criterion		− 7.196493
Log likelihood	44.37650	Hannan–Quinn criter		− 7.432388
F-statistic	9290.496	Durbin–Watson stat		1.931586
Prob(F-statistic)	0.000000			

#### Total social benefits

**Table:**

Variable	Coefficient	Std. error	t-Statistic	Prob.
Dependent variable: LOG(SOCIALBENEFIT)				
C	0.943916	0.870447	1.084404	0.3141
LOG(HEALTHEXP + PENSIONS)	0.854831	0.110111	7.763394	0.0001
R-squared	0.895942	Mean dependent var		7.698820
Adjusted R-squared	0.881077	S.D. dependent var		0.214956
S.E. of regression	0.074128	Akaike info criterion		− 2.172915
Sum squared resid	0.038465	Schwarz criterion		− 2.129088
Log likelihood	11.77812	Hannan–Quinn criter		− 2.267495
F-statistic	60.27028	Durbin–Watson stat		1.155281
Prob(F-statistic)	0.000110			

Subsidies.

**Table:**

Variable	Coefficient	Std. error	t-Statistic	Prob.
Dependent variable: LOG(SUBSIDIES)				
C	− 10.12436	2.417864	− 4.187317	0.0030
LOG(GDPP)	1.608420	0.252577	6.368047	0.0002
R-squared	0.835228	Mean dependent var		5.270283
Adjusted R-squared	0.814632	S.D. dependent var		0.315181
S.E. of regression	0.135699	Akaike info criterion		− 0.979892
Sum squared resid	0.147315	Schwarz criterion		− 0.919375
Log likelihood	6.899461	Hannan–Quinn criter		− 1.046279
F-statistic	40.55202	Durbin–Watson stat		1.217621
Prob(F-statistic)	0.000216			

Other expenditures.

**Table:**

Variable	Coefficient	Std. error	t-Statistic	Prob.
Dependent variable: LOG(EXPOTHER)				
C	− 1.740164	3.564512	− 0.488191	0.6385
LOG(REVTOTAL)	0.904533	0.416656	2.170933	0.0617
R-squared	0.370720	Mean dependent var		5.996880
Adjusted R-squared	0.292060	S.D. dependent var		0.243019
S.E. of regression	0.204474	Akaike info criterion		− 0.159895
Sum squared resid	0.334477	Schwarz criterion		− 0.099378
Log likelihood	2.799474	Hannan–Quinn criter		− 0.226282
F-statistic	4.712951	Durbin–Watson stat		2.203004
Prob(F-statistic)	0.061729			

## Identities

Lforce = partrate  *  pop_1564/100

un = lforce−emp

ur = un/lforce * 100

lforcelfs = emplfs + unlfs

urlfs = unlfs/lforcelfs * 100

pension_rate = crpension_ee + crpension_er

health_rate = crhealth_ee + crhealth_er

un_rate = crun_ee + crun_er

pensioncontr = 12 * gwage * pension_rate/100

healthcontr = 12 * gwage * health_rate/100

uncontr = 12 * gwage * un_rate/100

accidentcontr = 12 * gwage * craccident/100

soccontr = pensioncontr + healthcontr + uncontr + accidentcontr

avpension = pension_reprate * nwage/100

ydp = gdpp—taxes—soccontr + pensions

yd = ydp/cpi * 100

gdp = gdpp/pgdp * 100

grgdpfbih = @pcy(gdpp)

grgdp = @pcy(gdp)

inflation = @pcy(cpi)

prod = gdp/emp * 100

taxindirother = indirtaxrate * gdpp/100

taxindir = vat + taxindirother

taxes = taxindir + taxincpers + taxinccorp + taxother

revgross = taxes + contpension + conthealth + contun + revother

pensions = avpension * 12 * pensioners/1000000

exptotal = subsidies + socialbenefit + govcons + expother

govbal = revtotal  − exptotal

balgdp = govbal/gdp * 100

emp_high = emp − emp_low − emp_med

## List of variables

### Endogenous variables

ACCIDENTCONTR: Contributions to accident insurance
AVPENSION: Average monthly pension in KM
BALGDP: Budget balance in FBiH in relation to nominal GDP
CONTHEALTH: Contribution to health fund, mill. KM
CONTPENSION: Contribution to pension fund in mill. KM
CONTUN: Contributions for unemployment insurance in mill. KM
EMP: Total employment
EMP_HIGH: Employment high qualification
EMP_LOW: Employees with low qualification
EMP_MED: Employment medium qualification
EMPLFS: Total employment, LFS
EXPOTHER: Other expenditures, FBiH, mill. KM
EXPTOTAL: Total public expenditures of FBiH, mill. KM
GDP: GDP in FBiH, constant prices
GDPP: GDP in FBiH, current prices
GFCFP: Gross fixed capital formation in FBiH, current prices
GOVBAL: Budget balance in FBiH
GOVCONS: Public consumption, fiscal statistics, FBiH, mill. KM
GRGDP: Real GDP growth rate, FBiH
GRGDPFBIH: Nominal GDP growth rate, FBiH
GWAGE: Average gross wage per month in KM
HCP: Household consumption in FBIH, current prices, mill. KM
HEALTH_RATE: Total health insurance contribution rate
HEALTHCONTR: Contributions to health insurance
HEALTHEXP: Total expenditures of health fund
HEALTHREV: Total revenues of health fund
INFLATION: CPI inflation in FBiH
LFORCE: Labour force in FBiH
LFORCELFS: LFS labour force in FBiH
MP: Imports of FBiH, current prices, mill. KM
NWAGE: Average net salary per month in KM
PENSION_RATE: Total pension fund contribution rate
PENSIONCONTR: Contributions to pension insurance
PENSIONERS: Number of pensioners - 31.12
PENSIONS: Funds for the payment of pensions
PGDP: GDP deflator for BIH, assumed to be identical also for FBiH
PROD: Total nominal labour productivity, FBiH
REVGROSS: Public revenues—gross collection
REVOTHER: Other revenues
REVTOTAL: Total public revenues of FBiH, net collection, mill. KM
SOCCONTR: Social security contributions
SOCIALBENEFIT: Total social benefits, FBiH, mill. KM
SUBSIDIES: Subsidies, FBiH, mill. KM
TAXES: Tax revenues
TAXINCCORP: Corporate income tax—10%
TAXINCPERS: Personal income tax—10%
TAXINDIR: Revenues from indirect taxes
TAXINDIROTHER: Other indirect taxes (Revenues indirect tax—VAT)
TAXOTHER: Other taxes
UN: Unemployed—(number) average
UN_RATE: Total unemployment insurance contribution rate
UNCONTR: Contributions to unemployment insurance
UNLFS: Unemployed—(number) average, LFS
UR: Unemployment rate
URLFS: LFS unemployment rate
VAT: VAT
XP: Exports of FBiH, current prices, mill. KM
YD: Disposable income of private households (real)
YDP: Disposable income of private households (current prices), BiH
CRACCIDENT: Contribution rate to protect the natural and others. Accident

### Exogenous variables

CRCHILD_RS: Child protection fund contribution rate in RS
CRHEALTH_EE: Contribution rate for health. insurance (employee)
CRHEALTH_ER: Contribution rate for health. insurance (employer)
CRHEALTH_RS: Health fund contribution rate in RS
CRPENSION_EE: Contribution rate for the pension fund (employee)
CRPENSION_ER: Contribution rate for the pension fund (employers)
CRPENSION_RS: Pension fund contribution rate in RS
CRUN_EE: Contribution rate for unemployment insurance (employee)
CRUN_ER: The contribution rate for unemployment insurance (employer)
CRUN_RS: Employment fund contribution rate in RS
D2003: Dummy, 1 in 2003
D2006: Dummy, 1 in 2006
D2007: Dummy, 1 in 2007
D2008: Dummy, 1 in 2008
D2009: Dummy, 1 in 2009
D2010: Dummy, 1 in 2010
D2013: Dummy, 1 in 2013
HWWI: HWWI commodity price index, Euro basis
INDIRTAXRATE: Average indirect tax rate other than VAT (w.r.t. nominal GDP)
INTRATE: Long-term interest rates on loans to companies (BiH)
PARTRATE: Labour force participation rate
PENSION_REPRATE: Pension replacement rate
PERSINCTAXRATE: Personal income tax rate
POP_1564: Population aged 15 to 64
POP_6569: Population aged 65 to 69
POP_70PLUS: Population aged 70 plus
VATRATE: Value added tax rate
WORLDDEMAND: Sum of GDP in Germany, Austria, Italy, Slovenia, Croatia, Serbia, Montenegro, USA

## Footnotes

1 Draft Law on Contributions FBIH and Law on Income Tax, House of Peoples FBIH, August 2022.

## References

[CR1] Auerbach AJ, Gorodnichenko Y, Alesina A, Giavazzi F (2013). Fiscal multipliers in recession and expansion. Fiscal policy after the financial crisis.

[CR2] Batini N, Eyraud L, Forni L, Weber A (2014). Fiscal multipliers: size, determinants, and use in macroeconomic projections. IMF Tech Notes Man.

[CR3] Berg TO (2015). Time varying fiscal multipliers in Germany. Rev Econ.

[CR4] Blanchard O, Leigh D (2013). Growth forecast errors and fiscal multipliers. Am Econ Rev.

[CR5] Bouakez H, Chihi F, Normandin M (2014). Measuring the effects of fiscal policy. J Econ Dyn Control.

[CR6] Čapek J, Crespo Cuaresma J (2020). We just estimated twenty million fiscal multipliers. Ox Bull Econ Stat.

[CR7] Coenen G, Mohr M, Straub R (2008). Fiscal consolidation in the euro area: long-run benefits and short-run costs. Econ Model.

[CR8] Coenen G, Straub R, Trabandt M (2013). Gauging the effects of fiscal stimulus packages in the Euro area. J Econ Dyn Control.

[CR9] Cogan JF, Cwik T, Taylor JB, Wieland V (2010). New Keynesian versus Old Keynesian government spending multipliers. J Econ Dyn Control.

[CR10] Dufrénot G, Jambois A, Jambois L, Khayat G (2016). Regime-dependent fiscal multipliers in the United States. Open Econ Rev.

[CR11] Erceg CJ, Lindé J (2013). Fiscal consolidation in a currency union: spending cuts vs. tax hikes. J Econ Dyn Control.

[CR12] IMF (2008) World economic outlook, Chapter 5. Washington

[CR13] Lucas R, Brunner K, Meltzer A (1976). Econometric policy evaluation: a critique. The Philips curve and labor markets, Carnegie-Rochester conference series on public policy 1.

[CR14] Lucas RE (2003). Macroeconomic priorities. Am Econ Rev.

[CR15] Romer CD, Romer DH (2010). The Macroeconomic effects of tax changes: estimates based on a new measure of fiscal shocks. Am Econ Rev.

[CR16] Sargan JD, Hart PE, Mills G, Whitaker JK (1964). Wages and prices in the United Kingdom. A study in econometric methodology. Econometric analysis for national economic planning.

[CR17] Sargent T (1981). Interpreting economic time series. J Polit Econ.

[CR18] Sims E, Wolff J (2018). The Output and welfare effects of government spending shocks over the business cycle. Int Econ Rev.

[CR19] Spilimbergo A, Symansky S, Blanchard O, Cottarelli C (2009). Fiscal policy for the crisis. Cesifo Forum.

[CR20] Taylor JB (2009). The lack of an empirical rationale for a revival of discretionary fiscal policy. Am Econ Rev.

[CR21] Weyerstrass K (2009). A macroeconometric model for Bosnia and Herzegovina. East Eur Econ.

[CR22] Weyerstrass K, Neck R, Blueschke D, Majcen B, Srakar A, Verbič M (2020). Stabilisation policies in a small euro area economy: taxes or expenditures? A case study for Slovenia. Int J Comput Econ Econom.

